# ‘Treat my whole person, not just my condition’: qualitative explorations of hepatitis C care delivery preferences among people who inject drugs

**DOI:** 10.1186/s13722-021-00260-8

**Published:** 2021-08-12

**Authors:** Judith I. Tsui, Michael P. Barry, Elizabeth J. Austin, Elsa W. Sweek, Elyse Tung, Ryan N. Hansen, Michael Ninburg, John D. Scott, Sara N. Glick, Emily C. Williams

**Affiliations:** 1 Department of Medicine, Division of General Internal Medicine, University of Washington, Box 359780 - 325 9th Avenue, Seattle, WA 98104 USA; 2 Department of Epidemiology, University of Washington, Seattle, WA USA; 3 Department of Surgery, University of Washington, Seattle, WA USA; 4 Department of Health Services, University of Washington, Seattle, WA USA; 5 Center of Innovation for Veteran-Centered and Value-Driven Care, Health Services Research & Development, VA Puget Sound, Seattle, WA USA; 6 Department of Pharmacy, University of Washington, Seattle, WA USA; 7 Kelley-Ross Pharmacy Group, Seattle, WA USA; 8 Hepatitis Education Project, Seattle, WA USA; 9 Department of Medicine, Division of Allergy and Infectious Diseases, University of Washington, Seattle, WA USA; 10 HIV/STD Program, Public Health - Seattle & King County, Seattle, WA USA

**Keywords:** Hepatitis C virus, Substance-related disorders, Community pharmacy services, Direct-acting antivirals, Persons who inject drugs

## Abstract

**Background:**

The advent of direct-acting antivirals (DAAs)—a form of hepatitis C (HCV) treatment associated with shorter treatment course and greater efficacy—offers an unprecedented opportunity to eliminate HCV, but only if care delivery systems are developed to extend treatment to people who inject drugs (PWID). To support the design of a community-pharmacy program, we explored perspectives of PWID with chronic HCV with regard to barriers, motivators, preferences, and prior experiences related to HCV treatment and pharmacists.

**Methods:**

We conducted semi-structured interviews with people living with HCV who reported active injection drug use. Participants were recruited from local community service and clinical organizations in the Seattle, Washington region, and focus groups and interviews were conducted in-person or via phone/video-conference. Rapid Assessment Process was used to analyze qualitative data. Dual coders used structured templates to summarize findings and engaged in iterative review to identify themes.

**Results:**

Among the 40 participants, 65% were male, 52.5% were white, and 80% were not stably housed. On average, participants had been injecting drugs for 14 years and living with HCV for 6 years. Analyses revealed 3 themes: (1) limited knowledge regarding HCV and DAA treatments; (2) barriers/motivators for receiving treatment included fear of side effects, prior stigmatizing behaviors from physicians, and desire to protect relatives and the PWID community from HCV transmission; and (3) preferences for HCV care delivery, including a need for person-centered, low-barrier, and collaborative treatment integrated with other care (e.g. primary care and addiction treatment) for PWID. Participants were generally receptive to a community-pharmacy model for HCV treatment, but prior interactions with pharmacists were mixed and there were some concerns expressed that care delivered by pharmacists would not be equivalent to that of physicians.

**Conclusions:**

Even in the direct-acting antivirals era, people who inject drugs still face major barriers to hepatitis C treatment which may be reduced by providing low-barrier points of access for care through pharmacists. Key recommendations for community-pharmacy design included providing care team training to reduce stigma and ensuring care team structures and culture target PWID-specific needs for education and engagement.

**Supplementary Information:**

The online version contains supplementary material available at 10.1186/s13722-021-00260-8.

## Introduction

As a consequence of a national epidemic of opioid use disorders and injection drug use, including barriers to accessing needed harm reduction services, the incidence of hepatitis C virus (HCV) infection has been rising in parts of the world [1–3]. Injection drug use is the primary mode of HCV transmission, accounting for the majority of new infections in the United States (U.S.) [1–3] and other developed countries [4]. From 2010 to 2015 incidence rates increased by 167% nationally in the U.S. [5, 6], with particularly large increases among younger populations and in rural areas. National surveillance data of 34 states showed that the majority of states (88%) reported greater HCV incidence in young adults in 2012 compared to 2006, and incidence rose 13% per year in nonurban counties and 5% in urban counties [7]. Since 2013 deaths attributable to chronic HCV in the U.S. have surpassed all other infectious conditions (including HIV) combined [8], and HCV-related healthcare expenditures are projected to continue to rise [9]. Already, roughly 3 million Americans are estimated to have HCV [10] and 71 million are estimated to be infected world-wide [11]. If unaddressed, HCV will continue be a major cause of global morbidity and early mortality.

Encouragingly, the advent of direct-acting antivirals (DAAs) has greatly expanded our ability to treat and cure individuals living with HCV [12–15]. Compared to traditional, interferon-based therapies, DAAs offer several advantages, including greatly reduced side effects, shorter treatment duration (8–12 weeks compared to 6–12 months), and greater efficacy [16, 17]. Given the efficacy of DAAs and the accumulating evidence regarding the burden of untreated diseases, clinical guidelines now recommend treating all HCV-infected patients and prioritizing people who inject drugs (PWID) to interrupt forward transmission/incidence and reduce prevalence over time [18]. Modeling studies show that scale-up of HCV treatment among PWID is critical in achieving elimination [19], yet treatment gaps persist even in countries that provide coverage of medications for HCV [20]. A study of PWID living in Seattle, WA, which used data from the National HIV Behavioral Surveillance survey collected in 2018 (five years after DAAs became available) found that while the majority (> 90%) of HCV seropositive individuals reported being tested, less than 18% with HCV reported being treated and cured [21]. Traditional models of care relying on specialty referrals from primary care are not adequate for PWID. Prior research has demonstrated biases against treating active PWID among specialists [22]. PWID often lack regular primary care [23], instead frequently utilizing acute care clinics and emergency departments [24]. Models of HCV care integrated in addiction treatment settings are effective but do not reach PWID who are not seeking care for their addictions [25, 26]. Innovative community-based models are needed to achieve elimination goals in PWID.

A community pharmacy model for HCV care delivery holds potential to increase access to treatment/cure for PWID. Pharmacists have extensive clinical training, highly developed expertise in pharmacotherapy, and a long history of supporting management of acute and chronic diseases in healthcare settings [27, 28], making them ideally suited for the management of HCV. Pharmacies are ubiquitous, even in areas poorly served by physicians. In the U.S., 93% of people live within 5 miles of a community pharmacy [29]. Pharmacies have the added advantage of being easily accessible and able to provide flexible service without appointments. Through the mechanism of collaborative practice agreements (CPAs), pharmacists have authority to autonomously perform tasks related to medication management in collaboration with practitioners [30, 31], including in the context of overseeing DAA treatment. It allows pharmacists to perform certain functions that are delegated for specified circumstances under the agreement with the appropriate training. The scope of this authority varies state-to-state, but can include all aspects of medication management from testing and counseling to prescribing and dispensing medication for HCV treatment [32]. However, further exploration of the receptivity and preferences for such a model is needed, particularly with PWID and other populations who face barriers to primary care access.

Therefore, to support development of a community pharmacy-based model of HCV care for PWID, we conducted this qualitative study, rooted in ethnographic perspective, of active PWID who were living with HCV in Seattle, WA. Our goal was to explore PWID prior experiences with HCV care delivery, knowledge and attitudes towards HCV medications (specifically DAAs), and receptivity towards a community-pharmacy model of HCV treatment.

## Methods

Methods for recruitment, data collection and data analysis were guided by the Consolidated Criteria for Reporting Qualitative Research (COREQ) guidelines for reporting qualitative research [33].

### Study sample, recruitment, and eligiblity

Participants were recruited at syringe service programs, addiction treatment programs (e.g. methadone clinics and office-based buprenorphine programs), and a community based non-profit organization that provides services for persons with and at risk for HCV, as well as through flyers advertised at public locations frequented by PWID experiencing housing instability (e.g., libraries, shelters, public transit stops). Recruitment organizations were selected based on existing relationships with members of the study team. Participants were directly approached by study staff, referred by staff members at partner agencies, referred by other participants, or self-referred after having seen recruitment fliers at one of these agencies. When potential participants expressed interest, research staff briefly explained the study, obtained verbal consent prior to screening for eligibility, and enrolled eligible participants upon receipt of written informed consent. Participants were eligible if they: (1) were adults ≥ 18 years of age; (2) reported injection of any illicit substance within the past 90 days; (3) self-identified as having HCV but had not yet received treatment; and (4) were English speaking. We used a combination of purposive and snowball sampling approaches, with the goal of maximizing the diversity of our sample and thus reflecting the demographics of PWID in Seattle as closely as possible [34]. Initially, we utilized purposive sampling, which targeted the recruitment of participants with diverse demographic representation (e.g. sex, race/ethnicity) and diverse utilization of services for drug-related care (i.e. participants utilizing syringe service providers, office-based buprenorphine clinics, etc.). Later in the recruitment process, we leveraged snowball sampling to target harder to reach participants who were less accessible to the research team. Snowball sampling involved having existing study participants refer persons within their network that were not receiving services at the recruitment sites. This allowed the research team to recruit participants who did not have strong linkages to treatment programs or who experienced even more unstable or transient housing. Institutional Review Board approval was obtained from the University of Washington.

### Data collection

Once enrolled, participants completed either an individual interview or a focus group, depending on participant preference and availability. Each focus group occurred at a single recruitment setting and did not combine participants across settings, however, they did involve participants with a mix of demographic characteristics. Both interviews and focus groups were used to gather in-depth perspectives on HCV care delivery experiences as well as interactive discussion around preferences for a community-pharmacy model of HCV care. Data collection was conducted by a member of the study team (MB) trained in anthropology and qualitative methods.

Participants were asked to first complete a brief structured survey assessing their demographics, (gender, age, and race/ethnicity), housing status, duration and frequency of injecting drugs, substances recently injected, duration of awareness and degree of concern for being HCV-infected. Participants were then asked to respond to a series of open-ended questions framed by an interview guide based on domains from the Consolidated Framework for Implementation Research (CFIR) model [35]. The CFIR was used to guide interview development so that interviews could identify barriers and facilitators to program development and implementation. The guide was iteratively developed with input from members of the research team with qualitative research and content-specific expertise, and included open-ended questions related to (1) awareness and knowledge of new medications to treat HCV treatment and attitudes toward treatment; (2) experiences accessing HCV treatment; (3) preferences for HCV treatment (including timing, location of services, individual v. group/partners treatment); and (4) perspectives on receiving HCV treatment from pharmacists through a community-pharmacy program (see Additional file 1 for full interview guide). The research team reviewed the interview guide against the data after the first round of data collection was completed to further refine questions for clarity. Participants were recruited until data analysis suggested thematic saturation (targeted enrollment = 40).

Between 1/7/2020 and 7/17/2020, 40 participants enrolled and completed both the initial brief survey and qualitative interview (n = 24) or focus group (n = 16 participants, via 4 focus groups) with a research team member (MB). During January–February, data collection occurred in person at the respective recruitment site. After March 3, 2020, all research procedures including enrollment, informed consent, and data collection, were converted to remote encounters (phone or video) due to the COVID-19 pandemic. Interviews and focus groups took approximately 30–60 min to complete and were digitally recorded and professionally transcribed. Participants were reimbursed with a $40 gift card or cash.

### Data analysis

We characterized the sample using data from the short quantitative survey. We tabulated frequencies and proportions for categorical data, and means, medians, interquartile ranges (IQR), and standard deviations for continuous data.

The Rapid Assessment Process (RAP)—an intensive, team-based combination inductive/deductive approach to qualitative inquiry that uses triangulation and iterative data analysis to develop understanding [24, 36–39]—was used to analyze qualitative transcripts. This process is more accessible and efficient than traditional qualitative methods, can produce more actionable findings and recommendations, and is comparable to traditional qualitative analysis methods with approximately 80% overlap in findings [40]. Additionally, this approach was well-suited for this project in which qualitative data were needed to rapidly inform program design.

Two independent coders (MB and EA) reviewed and summarized transcripts using a structured analysis template in Microsoft Word [36, 37]. Templates were organized around a priori themes from the interview guide, and revised to include to emergent themes as analysis unfolded [35]. Templates were then iteratively reviewed by the qualitative study lead (EW) to ensure alignment and resolve discrepancies. Final templates were used to generate and refine key learnings and identify themes with core qualitative investigators (MB, EA, EW and JT).

## Results

Characteristics of participants are presented in Table 1. The median age was 37 (range 24 to 58), nearly one third were women, nearly half non-white, and the majority (80%) of the sample were not stably housed. Most study participants reported injecting daily and the most commonly reported drugs were heroin alone, or heroin and methamphetamine mixed together (i.e. “goofball”). The median duration of injecting drugs was 12 years (IQR: 5, 18), while the median length of time participants were aware of their HCV diagnosis was 2 years (IQR: 6 months, 7 years).

**Table 1:** Demographic and substance use characteristics of the sample of persons who inject drugs

	Overall (n = 40)
Age (Median, IQR)	37, 32–45
Gender	
Man	26 (65%)
Woman	13 (33%)
Non-binary (assigned male at birth)	1 (3%)
Race	
American Indian/Alaska Native	7 (18%)
Asian	1 (3%)
African American/Black	1 (3%)
White	21 (53%)
Multiple races	5 (13%)
Other race(s)	5 (13%)
Hispanic ethnicity	7 (18%)
Housing status	
Stably housed	8 (20%)
Not stably housed	32 (80%)
Drug(s) used at last injection episode	
Heroin, alone	14 (35%)
Methamphetamine, alone	4 (10%)
Heroin and methamphetamine	15 (38%)
3 or more drugs in combination	7 (17.5%)
Days injected in past month (Median, IQR)	30, 25–30
Injecting episodes on an average day (Median, IQR)	4, 3–4
Years injecting drugs (Median, IQR)	11.5, 5–18
Years aware of HCV diagnosis (Median, IQR)	2, 0.5–7

Twenty-four participants opted for individual interviews; the remaining 16 participated in one of four focus groups—one with eight participants and the rest with two or three participants. Qualitative analysis identified 3 themes, which are summarized below supported by quotes reflecting prototypical examples.

### Theme 1: Limited knowledge of HCV and DAA treatment

When asked about their knowledge of HCV, participants articulated gaps in knowledge about HCV transmission and low confidence in understanding how they became infected with HCV. As evidenced by this quote, there was uncertainty about sexual transmission risk relative to risks from injecting drugs.

“I’m not really sure if my ex-girlfriend has it or not because I couldn’t get a straight answer out of her if she had it or not. So I’m not really sure how I got it... they said it’s not really sexually transmitted, which kind of blew my mind, so I guess it might have been from shooting up or something..” [H010]

Participants also had limited understanding of HCV’s progression and physiological effects. Several noted feeling no different than “normal” while not clearly articulating an understanding that chronic HCV is generally asymptomatic until there is development of substantial liver injury/disease over time.

“I have personally haven’t noticed anything. I was diagnosed about a year ago and had a very little something, a little count or something was way under what it should have been. But I still haven’t noticed really anything that I can put my finger on.” [H021]

“If no one told me I had hepatitis C, I would have no idea.” [H022]

When asked about their familiarity with HCV treatment types, most participants had some knowledge of and strong negative feelings about interferon-based treatment regimens. Participants were much less aware of the existence of DAAs and the differences between interferon and DAA treatment regimens.

“If you told me that I had to go through interferon again, I would die with Hep C because, fuck no, I would never do that again. It was absolutely nightmarish.” [H009]“I know that the interferon one can be real hard on your liver and could almost be worse than just living with it. So that kind of was a turnoff for me.” [E002]

”

However, participants had a more positive outlook for the potential benefits of DAA treatment.

“I just heard it was a pill that you just take once a day. And it’s nothing like the interferon or whatever. There’s no side effects that make you sick or anything like that...It’s been nothing but positive, like results, as far as I’ve been told.” [X009]

“I know it’s not the interferon stuff, which is the really painful pain-in-the-ass process. I know it’s a three-month, once-a-day pill thing and it is not painful. And it should completely rid your body of hepatitis.” [X010]

Participants did voice concerns and uncertainty surrounding their limited knowledge of DAA side effects. A few participants also expressed concern over potential interactions between DAAs and other medications they were taking.

“Well, I don’t know the side effects. I don’t think there is any, but I’m not sure. I’m in a camper; I don’t want to be real sick in a camper. . .I don’t know, I just, I’m afraid. I don’t want to get sick.” [E005]

“… Because I've read or seen that even some of that stuff [medications for opioid use disorder] impacts some of the treatments that are out there. Like, has adverse reactions sometimes. Not with everybody, but it can. And sometimes that's what I worry about, too…” [X001]

### Theme 2: Barriers to and motivators for engaging in DAA treatment

Participants shared a number of common barriers to HCV treatment. Stigma regarding HCV was a key barrier, which participants experienced at multiple levels. To start, participants expressed feelings of shame (internalized stigma) connected to having a disease associated with and most often transmitted by injection drug use. In certain cases, participants reported that this sense of shame or internalized stigma led to avoidance of disclosure of HCV.

“I’m very ashamed of it. It’s like one of those things you don’t really want to talk about. It hurts because you – how I got Hep C was because I was sick and I needed to get well and I didn’t care that the dude had Hep C. I was hurting that bad and that’s why I’m ashamed of it because I took a chance.” [H008]

“Specifically with hepatitis, because it’s the connotation, too, that you are using needles, which you’re using drugs. It’s not like you said to somebody that you’re diabetic or that you have some type of cancer or some type of disease or illness that was hereditary or whatever.” [H012]

“I was staying with my mom because I’d been getting sick off and on but we couldn’t figure out what the heck it was. Because I was stubborn and wouldn’t go to the doctor. And I said it was a hangover or whatever, you know. And my mom said, I was turning yellow. I had jaundice in my eyes and stuff. So she took me and we found out what it was. Of course, the doctor told her the only way you really get it is through shooting up. It broke my mom’s heart. She just didn’t understand it.” [X002]

Additionally, participants shared experiences of interpersonal stigma via overt experiences including feeling judged by their physicians, which negatively impacted the experience of seeking HCV treatment and remaining engaged in care.

“You’re not supposed to judge your patients. And so many doctors in this world do judge their patients. . . . You’re supposed to understand them, not judge them, and treat them bad because they have a disease of the brain.” [E003]

“A lot of times because of how we look we’re perceived to be doing something wrong or illegal. […] even if you’re not.” [Unidentified focus group participant]

Yet another participant expressed the need for providers to practice without judgement, saying providers need to be “somebody that’s real, that don’t say one thing and do another, that doesn’t say, ‘Oh, she’s so fucked up. She’s done this and’- I get that enough. I don’t want to be judged no more.” [E005].

Numerous participants described experiences of having HCV treatment withheld because they were using substances, further demonstrating the role of interpersonal stigma in hindering HCV care engagement.

“My primary care said that I had to get clean to get the hepatitis treatment. I really didn’t try to do anything else about it after that. I was just like, all right, I’ll just live with it. I don’t want to get clean.” [H011]

“My actual doctor told me she wouldn’t treat me until I was not using anymore, because I was too high of a risk for getting reinfected. . . . I thought that was kind of, you know, a little biased, I guess, or whatever.” [X009]

Another barrier frequently cited by participants was the experience of unmet basic needs, including lack of shelter and food. Participants felt an imperative to prioritize basic needs over HCV care, and this discouraged their interest in HCV treatment. In some cases, the perpetual experience of unmet basic needs overwhelmed participants and led to a sense of nihilism/pessimism about the value of HCV treatment and health services in general.

“So if I had to choose between getting something to eat, getting food for my dog, drugs, and that pill, obviously dog food stays on top of the list. Then I’m going to go with drugs. And then I still got two choices to make at the end, and I don’t have time to do.” [H024]

Another participant shared frustration that “until I get stable housing, it’s just almost impossible” to maintain a medication regimen [H005]. This participant went on to say:

“I could care less if I get treated because I don’t care about another 20 years right now. For me, […] life is miserable and meaningless. And until I can get some type of normalcy back into my life, like I had when I was younger, I'll continue to be in this downward spiral of depression. I just don't see things getting much better for me.” [H005]

Furthermore, with many competing needs and ongoing substance use, there was fear of failure of getting and staying cured of HCV, as well as the burden of HCV treatment cost, despite DAAs being covered by most insurers in Washington State (including Medicaid).

“Well… it’s just me, I don’t have help. It’s just me and I already have so much on my plate. I don’t want to set myself up for failure again maybe.” [E005]

“I know the cost of it; it can be something around $80,000. And I’m afraid of getting cured of hepatitis and then reinfecting myself; thus, completely destroying the whole purpose of getting the treatment in the first place.” [X010]

Despite these barriers, participants also shared a variety of motivators for receiving treatment, including an altruistic desire to prevent transmission to others.

“My main concern is that when I am injecting, I’m afraid of spreading it to other people. So in my life, that’s what it seems to affect me the most, is concern about spreading it to other people.” [X010]

“I want to get rid of it because I do not want to give it to someone else… I actually want to get treated not for me, well yes, for me, but more along the lines of I don’t want to spread the disease.” [H009]

Participants highlighted the potential for PWID community to be a positive force for engagement in HCV treatment, providing awareness of treatment programs and providers through “word-of-mouth”.

“A lot of people don’t know where to go. If you show them, then they can show someone else, and show someone else.” [X005]

“So if we have a clinic where we see other people going in– and mainly it's hearsay and your friend. If it works for your friend or family member. . . or church member, . . . somebody you talk to and know, if they say, "Hey, this really works, look at me, do this, this and that…” … Seeing is believing.” [X002]

### Theme 3: Desire for integrated, flexible, and PWID-centered care delivery approaches

Participants shared preferences related to HCV care delivery, preferring a person-centered, holistic program that involves them in treatment planning and shared decision-making.

“Treat my whole person, not just my condition.” [H009]

“Everybody’s different, everybody has different needs. And everybody’s needs matter.” [H002]

Participants emphasized the importance of acknowledging the value of their lived experience with their illness as part of the treatment process.

“When it comes to questions about me, I’m the authority. But when it comes to questions about Hep C, they should be.” [H009]

“Because they would get to know me personally. They would know me. And I think that's huge; […] if somebody knew me, they would understand me more, I guess.” [E005]

Similarly, participants shared a preference for care delivery to involve individuals with personal, lived experience with HCV, as a mechanism to make care delivery more person-centered.

“I think it’s important to have people that have been through it working in places like this because the judgement factor… then when they’re telling you something, they’re not telling you because they have an agenda about it. They’re just telling it like it is… They literally are speaking from experience.” [Unidentified focus group participant]

“It really helps whenever they’re experienced users. And been there, done that. […] I like to hear their story. Gives you […] hope and motivation.” [X007]

Participants also expressed the importance of a low-barrier, flexible care delivery model with multiple opportunities to engage, and as few delays to HCV treatment as possible.

“[It’s] important that it is easy to get to, that you don’t have to go through a bunch of red tape and shit because that’ll – some people will quit right at the door… Less red tape saves lives in this situation.” [H024]

“. . .it needs to be really easy. You have lots of opportunities because even if the opportunity is right in front of [PWID], like if they need to go get high first, they’re going to go get high first. So I think if there’s multiple opportunities that’s right in front of them, they can get it better.” [H010]

Participants highlighted that providing flexibility of hours was a key opportunity to reduce barriers to PWID involvement in HCV care, especially for PWID who experience housing instability.

“I don’t have and hold a schedule that’s very consistent with the workday world… I start my day later, I’m up later. And so, a lot of the time when I’m starting my day, people are ending their day.” [H012]

Additionally, participants emphasized the benefit of co-locating HCV care with other services/care related to injecting drugs, as well as providing educational resources that could continue to engage PWID in needed services.

“Because most of the homeless or nomads out here, they’re not lazy, but they don’t like to go to different locations. If the location was a location where they normally all go… to get rigs and whatnot… I’m pretty sure that would attract a lot of people. [H023]

“Just having everything readily available. Mostly information is big or key. Everyone’s always wanting leaflets and pamphlets and more information.” [X005]

Overall, receptivity to the idea of a community-pharmacy model of HCV care delivery was mixed. Some participants believed such a model would provide a low-barrier point of entry for care for PWID.

“To just be able to walk into a pharmacy and say, “Hey, we want to get on this?” That would be interesting. … Drug users, we’re all about that satisfaction now. Anything to facilitate that.” [H021]

Several noted potential benefits of pharmacists’ specialized knowledge of medication interactions and side effects. For some participants, the community-pharmacy model was seen as a benefit because it gave PWID greater access to pharmacist expertise.

“I think a pharmacist might know a lot more about different medications than just a doctor. So you could ask him a lot more questions about what the effects and what it will do to you and how long it’d take. [H002]

“I have a blood-clotting disorder so I take anti-clotting medications. And so every time I go in and get something, the pharmacist also always makes sure that I don't have anything that's going counteract my drugs. […] They're extremely knowledgeable.” [X005]

Yet some participants voiced reservations about the model, which appeared to be rooted in lack of familiarity in the role, scope of practice, and level of training for pharmacists. Some participants viewed pharmacists as being markedly different than the medical providers they typically engaged with, and did not perceive pharmacists as equivalent to doctors.

“But I’m not going to a barber to get a New York strip steak. […] They’re a pharmacist. They’re administering a treatment a doctor prescribed. They’re not administering treatment themselves. […] If a pharmacist wanted to manage Hep C care, they should have become a doctor.” [H009]

Some participants described prior experiences with pharmacists, which were primarily transactional and, in some cases, negative. As a result of these experiences, some participants had negative attitudes towards pharmacists and hesitations about an HCV care delivery model that was driven by pharmacists.

“I don’t know. They’re not the doctor, they’re not the nurse… I’m not sure if I even would want them to be playing any of the roles. It’s hard to say… I don’t see them much different than the Walmart cashier at the front except they have a little more information about your medical records. So it’s difficult for me to say because they’ve typically been these sort of cold, almost burned out on their job kind of people.” [X010]

“I had this reoccurring problem where my prescription co-pay is like one dollar and I won’t have it, and it’s for something like antibiotics that I desperately need, and it’s really obvious that I need it because my whole face is peeling off at times. And the pharmacist won’t even help me out with a dollar.” [H005]

“You said pharmacy model. What came to mind is that you’d go to a pharmacy to get the treatment. I don’t like that. I like the availability of that being over the counter and the option of that, but for me I would need the support and structure of something like this. Just going to a pharmacy, they don’t like me there. The whole idea of the word pharmacy I don’t like. […] Those things I associate with negative stuff.” [Unidentified focus group participant]

## Discussion

This qualitative study of PWID with HCV provided important insights on barriers to and facilitators of HCV treatment in the DAA era, as well as preferences for care delivery to inform the development of a community pharmacy model. We found that participants experienced multiple barriers to receiving HCV treatment, including limited knowledge of the disease and its treatments, fear of medication side effects and drug interactions, physicians’ withholding treatment due to active substance use, stigma (both internalized and enacted by providers), competing needs (e.g. housing, food and substance use), and fear of failure of treatment and reinfection, while a primary facilitator was an altruistic desire to prevent transmission to others. We also learned that models of delivery of HCV care should be collaborative and patient-centered, and that they should also be “low-barrier” with flexible scheduling and multiple opportunities for engagement. These learnings echo the findings of other studies that have explored the perspectives of PWID and people experiencing homelessness related to receiving healthcare [41, 42], and reinforce the role of multi-level stigma as a major barrier to HCV care. The acceptability of a community pharmacy model for HCV among these PWID was mixed, and appeared in part influenced by a lack of familiarity with pharmacists’ training and scope of practice. Participants also described prior interactions with pharmacists that were transactional and not patient-centered, often contributing to their inability to receive needed care. As such, the learnings from this work indicate the need to provide community education around pharmacist credentials and capabilities, and the potential for newer pharmacist-based models of care to correct prior negativeexperiences.

This study contributes to the literature on barriers to DAA treatment for PWID with HCV in the U.S., and is, to our knowledge, the first qualitative study to explore the acceptability of a pharmacy-based model of care. Prior research has emphasized that providers face multiple barriers to providing HCV care via traditional models, including capacity, training, and access to resources (e.g. phleboltomy) [43–45]. These barriers call for the need for new models of care delivery to scale the provision of DAA therapies, particularly models that are decentralized, outreach-based, and involve peers [43]. Treating PWID for HCV is necessary to achieve national and global goals of HCV elimination, yet, as this study demonstrates, major barriers exist for this group that may not be overcome without new care delivery models [43, 45]. It should be noted that this study was conducted in 2020 (long after DAAs were first introduced in 2013) in Washington State, which currently has no restrictions on Medicaid coverage and is considered one of the best states in the U.S. for HCV medication access [46]. Yet such barriers are consistent with other research: a recent study of Seattle-area PWID found that only 18% reported being treated and cured of HCV [21]. A qualitative study by Madden et al. among PWID in Australia where there is universal coverage for DAAs similarly reported residual barriers, including a lack of urgency to seek treatment for HCV given its low symptomology, which participants in our study confirmed as well [42]. Thus, our study speaks to the growing evidence demonstrating that enacting policy-level changes to provide medication coverage is an insufficient strategy for PWID, and that implementation of care delivery models that are specifically targeted for this hard-to-reach population are needed. Prior research has demonstrated the feasibility and effectiveness of providing HCV treatment to less conventional settings such as primary care clinics [47–49], addiction treatment programs [50–53], and at harm reduction agencies/syringe service programs [54, 55]. Pharmacist-based models of care represent yet another important avenue for providing “de-centralized” and non-specialist dependent care.

Models of care delivery that utilize pharmacists and pharmacies are a promising strategy for expanding access to HCV treatment among PWID. A recent study by Radley et al. conducted in Scotland demonstrated effectiveness of a pharmacist-led program for HCV treatment for patients who were receiving treatment for opioid use disorder through community based pharmacies [56]. In the U.S., pharmacists have long been a key (and to a certain extent unrecognized) component of the Veterans Health Administration’s (VA) successful campaign to eliminate HCV [57]. Some successful examples of pharmacy-based programs outside of the VA also exist in the U.S. [58, 59], however, to our knowledge they have not been specifically tailored for PWID. Yet pharmacy-based programs have proven effective for other potentially life-saving medications for PWID. Expanded access to naloxone for overdose prevention among PWID in many states has occurred through pharmacy programs that utilize CPAs [60], and more recently, similar pharmacy-based programs have been utilized to provide pre-exposure prophylaxis (PrEP) for HIV among persons at risk [61, 62]. Pharmacy models have also been demonstrated to reduce barriers to care for people experiencing homelessness. Johnsen, et al. (2021), for example, found that not only was their outreach pharmacist model acceptable for people experiencing homelessness, it reduced barriers and further encouraged engagement in care by “capitalizing on windows of opportunity” that patients had to get treated [41].

**Fig. 1:**
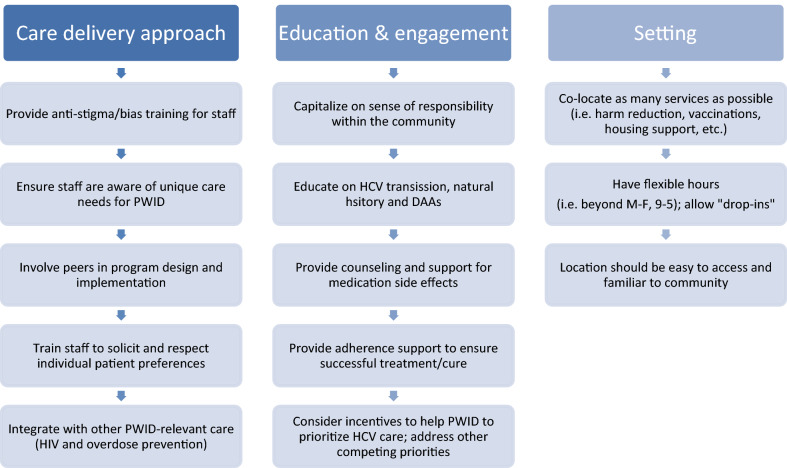
Care delivery model recommendations

Through the present study, specifically designed to inform our community-based pharmacy model of care delivery, we gained important insights into the pharmacy-based model we are developing and pilot testing. Specifically, though participants had limited experience with pharmacists, they were enthusiastic about the potential pharmacists hold in helping them understand DAA treatment and side effects, due to expertise in medications. They also expressed beliefs that pharmacies might provide a more “low-barrier” point of entry to care. It is notable that many of these PWID we interviewed recounted negative experiences with physicians where they felt stigmatized and had medications withheld due to their substance use, which then discouraged them from seeking further care from those settings and providers. Providing an alternative for HCV treatment through pharmacies may reach such patients who have been alienated by traditional models of care. An additional barrier to HCV treatment that may be addressed through a pharmacy model was the fear of treatment failure and reinfection. Although DAAs are highly efficacious, treatment failures can occur due to poor adherence, such as in the case of treatment interruptions, premature discontinuations due to side effects or failure to provide timely refills to patients. Pharmacists may have more time and training to provide adherence counseling, education and support on medication side effects than physicians, and they have experience navigating insurance requirements for authorization of medications. Furthermore, since they are also involved in dispensing medication, they can be aware of, and respond more quickly to, patient non-adherence as signaled by delays in refill pick-ups. As such, they are arguably uniquely positioned to help PWID fully adhere to HCV medication and prevent treatment failures. Pharmacies are also a site for needle/syringe procurement in many states, and can thus can assist patients in preventing reinfection. Yet this study also demonstrated that not all PWID were accepting of the idea that pharmacists could provide clinical care like doctors. As such, expanding the role of pharmacists to diagnose and treat conditions like HCV may first require educating PWID about pharmacists’ extensive training and accreditation requirements to reassure of their clinical competency. The learnings from this study will inform the design of our intervention with the goals of enabling access, reducing barriers, and addressing community-specific needs for care delivery approaches (Fig. 1).

There are limitations to this study. Interviews and focus groups were conducted among PWID with HCV who were recruited within Seattle; results may not be generalizable to other locations or communities. Although we used a combination of purposive and snowball sampling in order to maximize the diversity of our sample, certain populations may be under-represented (such as persons of color, incarcerated individuals, or persons with mobility issues). Due to the COVID-19 pandemic, later data collection was conducted remotely which could impact participants level of trust and willingness to disclose.

In summary, this qualitative research offers valuable insights related to HCV treatment barriers and preferences for future care delivery models, which allowed us to tailor our community pharmacy model to address the complex needs and preferences of PWID living with HCV. Our findings suggest that “low-barrier” programs that integrate other essential harm reduction services, provide care in a non-stigmatizing fashion, and consider PWID lived experiences, are needed given the multidimensional needs and barriers of the PWID population. Future research will test the feasibility and outcomes of such a community-pharmacy based model for HCV treatment tailored for PWID in Washington State.

## Supplementary Information

**Additional file 1.** Interview guide for semi-structured interviews and focus groups.

## Data Availability

The datasets used and/or analyzed during the current study are available from the corresponding author on reasonable request.

## Abbreviations

DAAs: Direct-acting antivirals
HCV: Hepatitis C
PWID: People who inject drugs
CPAs: Collaborative practice agreements
CFIR: Consolidated Framework for Implementation Research
RAP: Rapid Assessment Process
