# Supplementary material for: ‘Treat my whole person, not just my condition’: qualitative explorations of hepatitis C care delivery preferences among people who inject drugs
Source: Addict Sci Clin Pract. 2021 Aug 12;16:52. doi: 10.1186/s13722-021-00260-8 (PMC8358259; doi:10.1186/s13722-021-00260-8)
Supplement: Supplementary file 1 — Additional file 1. Interview guide for semi-structured interviews and focus groups. [file 13722_2021_260_MOESM1_ESM.docx]

**SUPPLEMENTAL MATERIAL A: Interview Guide for Semi-Structured Interviews and Focus Groups**

**Topic A:** *Experience, Knowledge, and Beliefs around HCV Treatment, and preferences for ideal setting/model*

1. Tell me about your experience living with hepatitis C.
2. What do you know, or have you heard about hepatitis C?
   1. Where have you gone to learn more about hepatitis C?
3. Have you ever sought help or treatment for your hepatitis C?
   1. Why or why not?
   2. What avenues felt open to you? What avenues felt closed to you?
   3. If you have, tell me about your experience.
   4. Was your hepatitis C treated?
   5. Can you tell me about things that were easy or not easy about getting this treatment?
4. What can you tell me about medications for hepatitis C?
   1. What have you heard about the new medications for HCV?
   2. What do you know about [DAAs]?
5. Describe an ideal treatment experience for your hepatitis C.
   1. What setting is treatment offered in?
      1. What makes this environment appealing to you?
   2. Who would you ideally want to get treatment from?
      1. What qualities make this individual appeal to you?

**Topic B:** *Additional Intervention for PWIDs*

1. Have you ever sought help for your opioid or substance use? If so, where did you go?
   1. What avenues felt open to you? What avenues felt closed to you?
   2. Can you tell me about things that were easy or not easy about getting this treatment?
2. What kinds of medications have you received to help you with opioid or other substance use?
   1. Tell me about your experiences with these medications.
3. Have you ever sought help to prevent overdose? If so, where did you go?
   1. What avenues felt open to you? What avenues felt closed to you?
   2. Can you tell me about things that were easy or not easy about getting this treatment?
4. Have you received medicines to prevent overdose?
   1. If so, what kind? How would you take them?
   2. Tell me about your experiences with them
5. The next few questions are about HIV prevention. Please feel free to speak about your own experiences and knowledge. Please do not use anyone’s name and do not disclose anyone’s HIV status.
6. Have you heard about medications that prevent HIV infection [PrEP]?
   1. *If no, give brief description of PrEP:*
      1. There is a method of HIV prevention called “PrEP.” Taking PrEP medications daily lowers a person’s chance of contracting HIV through sexual contact and injecting drugs.
7. *If yes,* what have you heard?
8. How would you describe your community’s knowledge about this HIV prevention medication?
9. How do you think those in your community would feel about taking a daily medication to prevent HIV?

**Topic C:** *HCV Treatment Preferences & Pharmacy-based approach*

1. What comes to mind when you think of a pharmacist, or a provider who administers medications?
   1. Can you describe a positive experience you’ve had with a pharmacist?
   2. Can you describe a negative experience you’ve had with a pharmacist?
   3. What expectations for pharmacists’ behaviors would you have?
2. If you were able to walk into a pharmacy to receive help with your hepatitis C- (and not have to see a doctor at another location first), would you be interested?
3. What kind of services/treatments might you want from a community-pharmacy setting through a pharmacist?
   1. What kinds of services/treatments might you not want from a community-pharmacy setting through a pharmacist?
4. Do you think your injecting partners would be interested in getting HCV treatment in a community pharmacy?
   1. Why? Why not?
5. Would you be interested in bringing injecting partners to be treated at the same time as you?
   1. Why? Why not?
6. Does anyone have additional thoughts or input about the questions I’ve asked today?
7. Is there anything else you’d like the study team to know, or think about as we move forward?

**Topic D** (added April, 2020)**: COVID-19 Impact on PWID**

1. What have you heard about COVID-19?
   1. Where are you getting information about COVID-19?
2. How has COVID-19 impacted your life?
   1. … the medical care/services you use in the area?
   2. … the ways you interact with your community?
   3. … the ways you take care of yourself?
   4. … the way you use drugs?
3. How has COVID-19 impacted your community’s life?
